# The Middle East Respiratory Syndrome Coronavirus (MERS-CoV) Does Not Replicate in Syrian Hamsters

**DOI:** 10.1371/journal.pone.0069127

**Published:** 2013-07-02

**Authors:** Emmie de Wit, Joseph Prescott, Laura Baseler, Trenton Bushmaker, Tina Thomas, Matthew G. Lackemeyer, Cynthia Martellaro, Shauna Milne-Price, Elaine Haddock, Bart L. Haagmans, Heinz Feldmann, Vincent J. Munster

**Affiliations:** 1 Laboratory of Virology, Division of Intramural Research, National Institute of Allergy and Infectious Diseases, National Institutes of Health, Hamilton, Montana, United States of America; 2 Division of Clinical Research, Division of Intramural Research, National Institute of Allergy and Infectious Diseases, National Institutes of Health, Frederick, Maryland, United States of America; 3 Department of Viroscience, Erasmus Medical Center, Rotterdam, The Netherlands; 4 Department of Medical Microbiology, University of Manitoba, Winnipeg, Manitoba, Canada; Faculty of Biochemistry Biophysics and Biotechnology, Jagiellonian University, Poland

## Abstract

In 2012 a novel coronavirus, MERS-CoV, associated with severe respiratory disease emerged in the Arabian Peninsula. To date, 55 human cases have been reported, including 31 fatal cases. Several of the cases were likely a result of human-to-human transmission. The emergence of this novel coronavirus prompts the need for a small animal model to study the pathogenesis of this virus and to test the efficacy of potential intervention strategies. In this study we explored the use of Syrian hamsters as a small animal disease model, using intratracheal inoculation and inoculation via aerosol. Clinical signs of disease, virus replication, histological lesions, cytokine upregulation nor seroconversion were observed in any of the inoculated animals, indicating that MERS-CoV does not replicate in Syrian hamsters.

## Introduction

In June of 2012, a novel coronavirus, designated Middle East respiratory syndrome coronavirus (MERS-CoV) [1] and classified as a 2c betacoronavirus [2,3], was isolated from a patient with a fatal case of pneumonia and renal failure in Saudi Arabia [3]. To date, 55 human cases of MERS-CoV have been reported with 31 fatalities [4], including two fatal cases in Jordan in April of 2012 that were retrospectively detected. A cluster of cases in the UK in February of 2013 suggested human-to-human transmission in two cases with no travel history to the Middle East [4].

Several 2c betacoronaviruses with high sequence identity to MERS-CoV have been detected in bats in Europe, Ghana and Mexico [5,6] suggesting that bats are the natural reservoir of MERS-CoV. It is currently unclear whether human cases were a result of direct zoonotic transmission from this reservoir to humans or whether an intermediate host was involved. In vitro data suggest that MERS-CoV has a broad host range [7], increasing the likelihood that an intermediate host was involved in amplifying or transmitting the virus from its natural reservoir to humans.

In 2003 another betacoronavirus, SARS-CoV, caused a pandemic with approximately 8000 human cases and a case fatality rate of ~10% [8]. The emergence of MERS-CoV with a high case fatality rate and the potential to transmit between humans stresses the need for a small animal model to study the pathogenesis of this virus and to test the efficacy of potential therapeutic or prophylactic intervention strategies. In Syrian hamsters, SARS-CoV replicates extensively in the respiratory tract in the first week after intranasal inoculation with 10^3^ TCID_50_ [9] and, depending on the SARS-CoV strain used, can cause mortality in a small subset of infected animals [10]. In the present study, we explored the suitability of the Syrian hamster as a small animal model for MERS-CoV isolate HCoV-EMC/2012 infection or disease.

## Materials and Methods

Ethics Statement. All animal experiments were approved by the Institutional Animal Care and Use Committee of the Rocky Mountain Laboratories, and performed following the guidelines of the Association for Assessment and Accreditation of Laboratory Animal Care, International (AAALAC) by certified staff in an AAALAC-approved facility.

The work with infectious MERS-CoV was approved under BSL3 conditions by the Institutional Biosafety Committee (IBC). Sample inactivation was performed according to standard operating procedures approved by the IBC for removal of specimens from high containment.

Virus and cells. MERS-CoV isolate HCoV-EMC/2012 was propagated in VeroE6 cells in Dulbecco’s Modified Eagle Medium (DMEM; Sigma) supplemented with 2% fetal calf serum (Logan), 1 mM L-glutamine (Lonza), 50 U/ml penicillin and 50 µg/ml streptomycin (Gibco). The HCoV-EMC/2012 virus stock was titrated by end-point titration in VeroE6 cells. VeroE6 cells were inoculated with tenfold serial dilutions of virus stock in DMEM supplemented with 2% fetal calf serum, 1 mM L-glutamine, 50 U/ml penicillin and 50 µg/ml streptomycin. Five days after inoculation, cytopathic effect (CPE) was scored and the TCID_50_ was calculated from 10 replicates by the method of Spearman-Karber.

Animal experiments. Three groups of 40 hamsters were inoculated with MERS-CoV isolate HCoV-EMC/2012; one group received 4x10^2^ TCID_50_ via aerosols (see below), one group received 10^3^ TCID_50_ intratracheally in a total volume of 300 µl, and one group received 10^6^ TCID_50_ intratracheally in a total volume of 300 µl. Mock infected hamsters (four animals per time point) inoculated with DMEM intratracheally or via aerosols were included as controls for histopathology and cytokine analysis. Eight hamsters in each group and eight control hamsters (4 intratracheal and 4 aerosol) were injected with an IPTT-300 temperature transponder (BMDS) to monitor body temperature daily. Animals were weighed daily and observed for signs of disease. Nasal, oropharyngeal, urogenital and rectal swabs were obtained on 1, 2, 3, 4, 5, 6, 7, 9 and 11 days post inoculation (dpi) and analyzed for the presence of viral RNA. On 2, 4, 8, 14 and 21 dpi, eight hamsters in each group were euthanized and trachea, heart, lung, spleen, liver, kidney, ileum, colon, bladder, nasal turbinates and brain were collected for virological and histopathological analysis.

Aerosol challenge. Hamsters were exposed to MERS-CoV isolate HCoV-EMC/2012 using a 38 liter, whole body exposure chamber within a Class II biological safety cabinet inside high containment. The animals received a single, 10 minute aerosol exposure and were contained using stainless steel, wire mesh cages (2 hamsters/cage). Anesthesia was not administered to the animals. The aerosol challenge consisted of 5 runs with each run containing 4 wire mesh cages. Viral inoculum and collection material were prepared the day of the exposure using DMEM and 1% fetal calf serum. Aerosol particles were generated by a 3-jet Collison nebulizer (BGI Inc., MA, USA) operating at 7.5 lpm (25-30 PSI) and ranged from 1–3 µm in size. The contents of the aerosol exposure were collected in an All Glass Impinger (AGI, Ace Glass Inc., NJ, USA) continuously operating at 6.0 lpm. Both generator and sampler were flow checked using a frictionless bubble meter (Gilibrator, Sensidyne LP, FL, USA) to ensure the desired flow rates and pressure were achieved. In between aerosol runs a period of 5 minutes allowed the particles from the previous run to settle. The exposure chamber maintained negative pressure throughout the duration of the inoculation. An aerosol management platform (AeroMP, Biaera Technologies, USA) was used to conduct the exposures. The automated aerosol system controls, monitors, and maintains several parameters that impact aerosol studies [11]. Respiratory minute volume rates were determined using a formula derived by Alexander et al [12]. Weights were averaged for aerosol exposure group/run (n=8, n=40 total). A presented dose was calculated using the simplified formula D = R x C_aero_ x T_exp_ [11], where D is the presented or inhaled dose (expressed in TCID_50_), R is the respiratory minute volume (L/min), C_aero_ is the aerosol concentration (TCID_50_/L), and T_exp_ is duration of the exposure (min).

Histopathology. Histopathology was performed on select hamster tissues. After fixation for 7 days in 10% neutral-buffered formalin and embedding in paraffin, tissue sections were stained with hematoxylin and eosin (H&E). For the histopathological analysis of the nasal turbinates whole hamster skulls were used. The skulls were decalcified using a 20% EDTA solution in sucrose (Newcomer Supply) and allowed to sit at room temperature for 3 weeks. The 20% EDTA/sucrose solution was changed once prior to mid-sagittal sectioning of the skull.

Immunohistochemistry. Immunohistochemistry was performed on lungs, kidney, small intestine, urinary bladder and colon of normal hamsters using an α-DPP4 antibody (α-CD26; 1:800; Abcam). The tissues were processed for immunohistochemistry using the Discovery XT automated processor (Ventana Medical Systems) with a DapMap kit (Ventana Medical Systems).

RNA extraction. RNA was extracted from swab samples using the NucleoSpin 96 Virus Core kit (Macherey-Nagel) and a Corbett Robotics model CAS 1820 automatic RNA extractor. RNA was eluted in 100 µl. RNA was extracted from whole blood using the QiaAmp Viral RNA kit (Qiagen) according to the manufacturer’s instructions. RNA was eluted in 60 µl. Tissues (30 mg) were homogenized in RLT buffer and RNA was extracted using the RNeasy kit (Qiagen). RNA was eluted in 50 µl.

Quantitative PCR. For detection of viral RNA, 5 µl RNA was used in a one-step real-time RT-PCR upE assay [13] using the Rotor-Gene^TM^ probe kit (Qiagen) according to instructions from the manufacturer. In each run, standard dilutions of a titered virus stock were run in parallel, to calculate TCID_50_ equivalents in the samples. Hamster Mx2 gene expression was determined as described previously [14]. qRT-PCR was performed as described above using Mx2 specific primers. The fold-change of each gene was calculated by normalizing the change in C_T_ (ΔC_T_) to the C_T_ values for RPL18 as an internal reference gene for each sample and comparing this to the C_T_ values of mock inoculated hamsters (2^-ΔΔCT^).

ELISA. Immuno-globulin G antibody responses were measured in an enzyme-linked immunosorbent assay (ELISA) using MERS-CoV isolate HCoV-EMC/2012. HCoV-EMC/2012 containing cell culture supernatant was concentrated and purified by spinning two hours at 21000 rpm over a 15% OptiPrep (Axis-Shield) cushion. The pellet was resuspended in PBS and triton X-100 was added to a final concentration of 1% and the suspension was then gamma-irradiated before removal from high containment. This suspension was then used to coat immuno 96 microwell maxisorp plates (NUNC) at 4°C overnight. Subsequently, plates were blocked with 5% skim milk in PBS containing 0.05% Tween 20 (PBST) for 1.5 hours at 4°C. After 3 washes with PBST, 50 µl of diluted serum samples were added, and the plates were incubated for 1 hour at 37°C. Bound antibodies were detected after 3 washes using an anti-hamster secondary antibody conjugated with horseradish peroxidase (HRP; KPL). Following incubation for 1 hour at 37°C, bound HRP was detected using the ABTS® Peroxidase Substrate System (KPL). The absorbance at 405 nm was measured using a microplate spectrophotometer. Sera were considered positive when absorbance was higher than three standard deviations above the mean of negative control sera. Sera obtained from rabbits immunized with HCoV-EMC/2012 were used as a positive control.

## Results & Discussion

**Figure 1:**
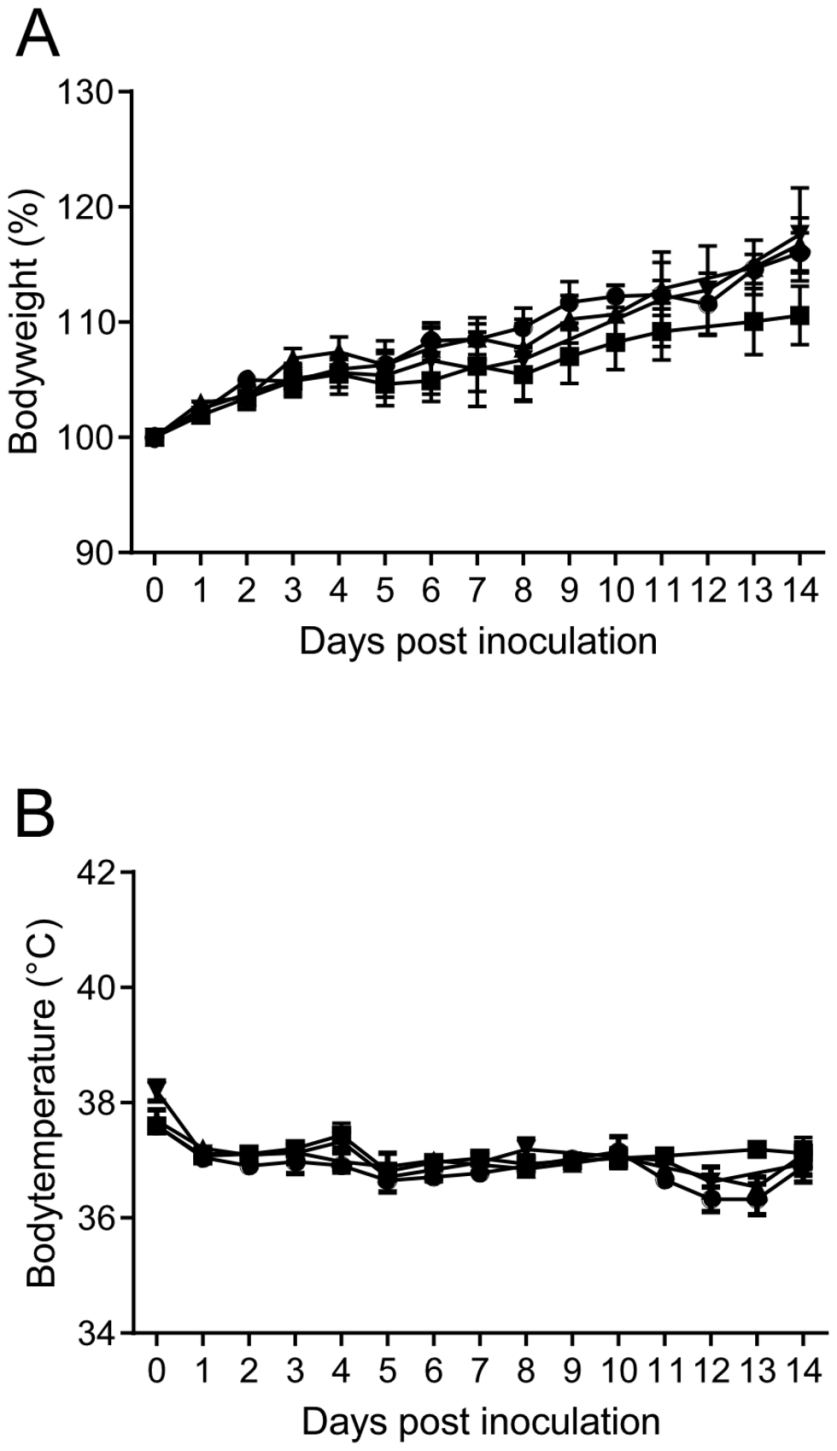
Body weight and temperature in Syrian hamsters inoculated with MERS-CoV isolate HCoV-EMC/2012.

**Figure 2:**
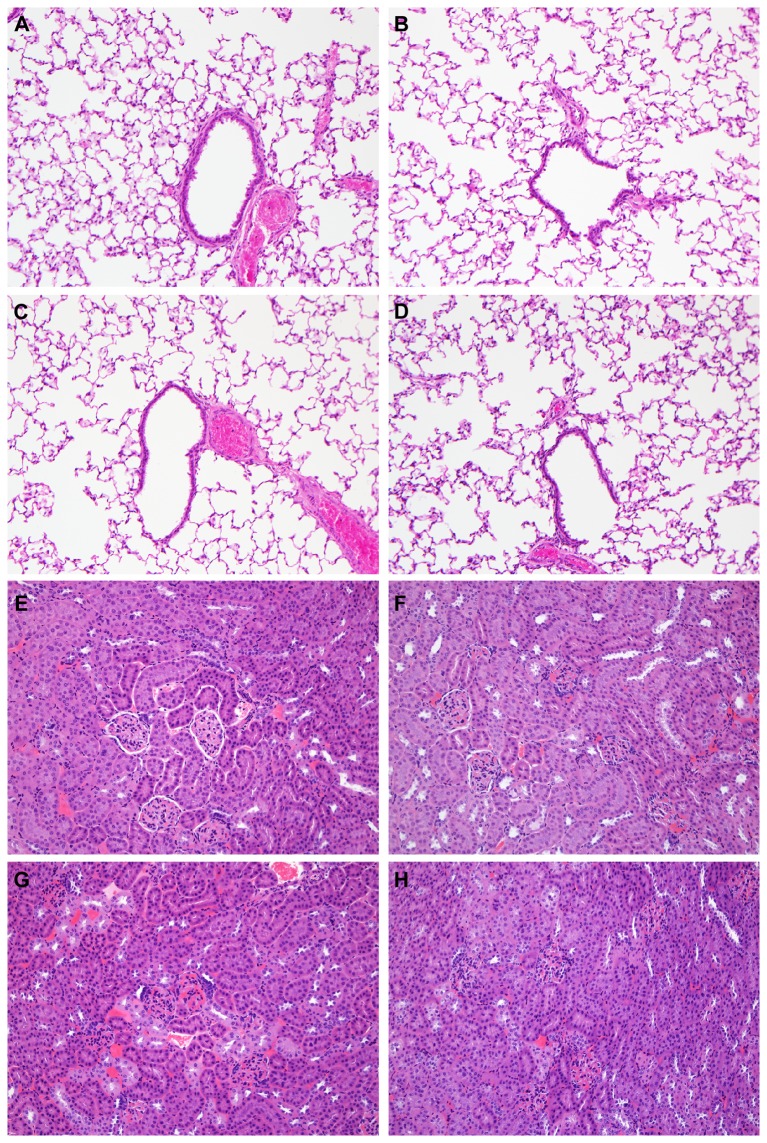
Histological analysis of lungs and kidney of Syrian hamsters inoculated with MERS-CoV isolate HCoV-EMC/2012.

Hamsters were divided into 3 groups of 40 hamsters. The first group was inoculated intratracheally with 10^3^ TCID_50_ of MERS-CoV isolate HCoV-EMC/2012; the second group was inoculated intratracheally with 10^6^ TCID_50_ of HCoV-EMC/2012 and the third group was inoculated with 4x10^2^ TCID_50_ HCoV-EMC/2012 via aerosolization. Hamsters were observed for clinical signs of disease daily for 21 dpi and body weight and body temperature were measured. None of the hamsters in the three inoculated groups showed signs of disease, weight loss or increased body temperature (Figure 1). Nasal, oropharyngeal, urogenital and rectal swabs were obtained daily between 1 and 11 dpi and were all negative by qRT-PCR. Upon necropsy on 2, 4, 8, 14 and 21 dpi, no gross lesions were observed. Lungs, spleen and mandibular lymph nodes collected on 2, 4 and 8 dpi were analyzed for the presence of HCoV-EMC/2012 vRNA by qRT-PCR and found to be negative. Trachea, heart, lung, spleen, liver, kidney, ileum, colon, urinary bladder, nasal turbinates and brain collected on 2, 4, 8, 14 and 21 dpi were used for histopathological analysis; no lesions were observed that could be attributed to the virus in any of the tissues examined (Figure 2).

Hamsters were mock inoculated (●), inoculated intratracheally with 10^3^ TCID_50_ HCoV-EMC/2012 (■), inoculated intratracheally with 10^6^ TCID_50_ HCoV-EMC/2012 (▲) or inoculated with 4x10^2^ TCID_50_ via aerosols (▼) and body weight (A) and temperature (B) were measured. Average and standard error of the mean are plotted for 8 animals per time point.

Hamsters were mock inoculated (A and E), inoculated intratracheally with 10^3^ TCID_50_ HCoV-EMC/2012 (B and F), inoculated intratracheally with 10^6^ TCID_50_ HCoV-EMC/2012 (C and G) or inoculated with 4x10^2^ TCID_50_ via aerosols (D and H). On 4 days post inoculation tissue sections of the lungs (A–D) and kidneys (E–H) of these animals were collected and stained with hematoxylin-eosin. The panels shown in this figure are presentative for 4 animals analyzed per tissue per group.

**Figure 3:**
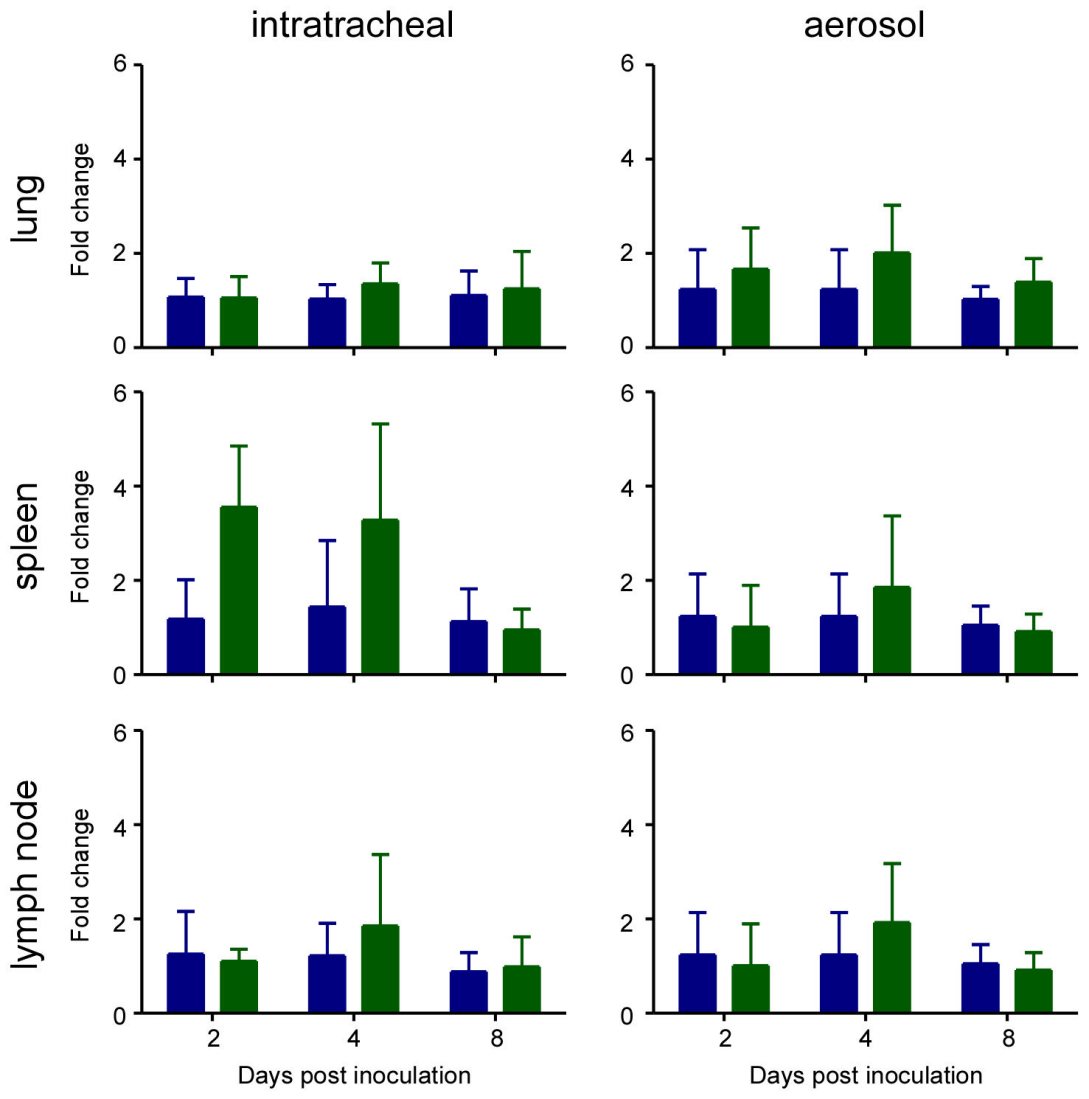
Mx2 gene expression in hamsters after inoculation with MERS-CoV isolate

**Figure 4:**
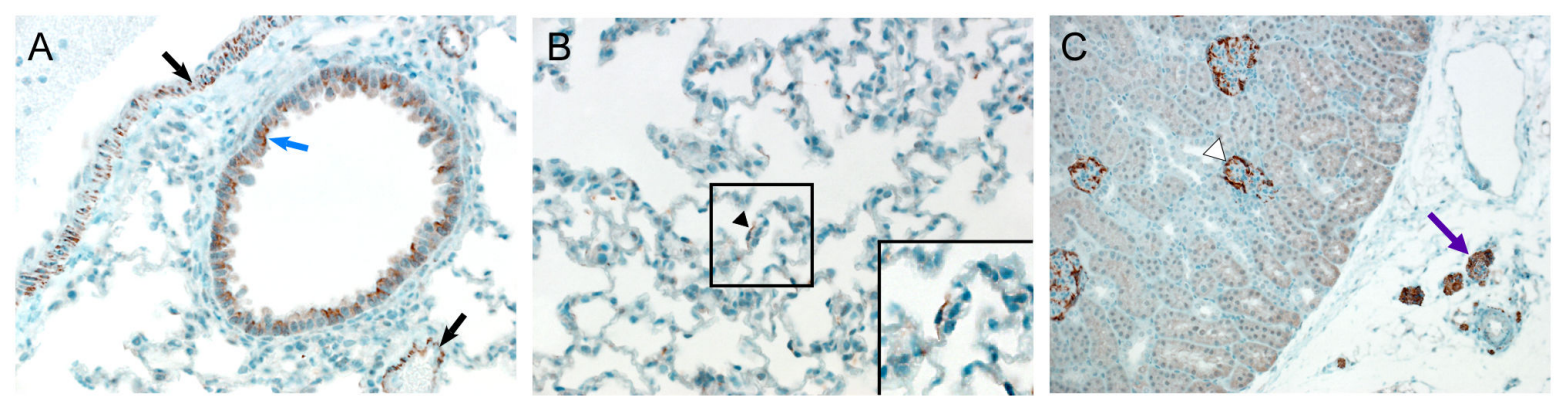
Presence of DPP4 in hamster lung and kidney.

Lungs, spleen and lymph nodes collected from the hamsters intratracheally inoculated with 10^6^ TCID_50_ and inoculated via aerosols on 2, 4 and 8 dpi were also analyzed for upregulation of the transcription of the Mx2 gene. Mx2 gene expression was analyzed as an indicator of an innate immune response to virus infection, since Mx2 is downstream of STAT1 and as such is an indicator of a type I or type III IFN response (reviewed in 15. A statistically significant upregulation of Mx2 gene expression could not be detected in any of the hamsters, independent of the inoculation route, inoculation dose or tissue analyzed (Figure 3). Taken together these data indicate that the hamsters were either not infected at all or the infection occurred below the limit of detection of our assays. To determine whether the animals were infected at all, we developed an ELISA assay to detect antibodies against MERS-CoV. Hamster sera collected on 21 dpi were analyzed for the presence of antibodies; there was no evidence for seroconversion in any of the inoculated animals, regardless of inoculation route or dose. Thus, the MERS-CoV does not replicate in Syrian hamsters and they are an unsuitable model for studies into pathogenesis or potential prophylactic or therapeutic intervention strategies. To determine whether the recently described receptor for MERS-CoV, DPP4 [16], is expressed in the Syrian hamster, we performed immunohistochemistry on lung, kidney, small intestine, urinary bladder and colon of normal hamsters using an α-DPP4 antibody. DPP4 could be detected in all tested hamster tissues (Table 1) and was abundantly present in the lungs and kidneys of Syrian hamsters (Figure 4). The abundant expression of DPP4 on bronchiolar epithelium and the occasional presence on type I pneumocytes indicates that the absence of replication in the Syrian hamster is not due to a lack of the receptor, but rather to the fact that the epitope that MERS-CoV binds to is not conserved in Syrian hamster DPP4 or that other restrictions exist on the cellular level that prevent virus replication.

HCoV-EMC/2012. A qRT-PCR assay to detect Mx2 was performed on RNA isolated from lungs, spleen and mandibular lymph nodes collected on 2, 4 and 8 days post inoculation (dpi) of Syrian hamsters with 10^6^ TCID_50_ of HCoV-EMC/2012 (green bars) via the intratracheal route (left panels) or 4x10^2^ TCID_50_ of HCoV-EMC/2012 via aerosol (right panels, green bars) and compared to mock inoculated animals (blue bars). Data are shown as the fold change of Mx2 over uninfected controls and normalized to an internal reference gene (RPL18). Means were calculated from 4 mock animals and 4 (2 dpi) or 8 (4 and 8 dpi) inoculated animals. Error bars represent standard deviation.

**Table 1:** DPP4 expression in hamster tissues as detected by immunohistochemistry.

	Lung	Kidney	Small intestine	Urinary bladder	Liver	Colon
Bronchiolar epithelium	+					
Type I pneumocyte	+					
Nerve	+	+	+	+	-	+
Arteriolar smooth muscle	+	+	-	-	-	-
Glomerular parietal epithelium		+				
Lamina muscularis			+			-
Lamina propria vessels			+			-

+ DPP4 detected; - DPP4 not detected. Open fields indicate the cell type is not present in this tissue.

Immunohistochemistry was performed on Syrian hamster lung and kidney tissues using an α-DPP4 antibody. In the lung (A and B), DPP4 was abundantly present on bronchiolar epithelium (blue arrow) and smooth muscle cells (black arrows) and was occasionally present on type I pneumocytes (black arrowhead and inset in panel B). In the kidney, DPP4 was present on glomerular parietal epithelium (white arrowhead) and in nerve tissue (purple arrow).

Although these experiments did not result in a small animal model for MERS-CoV, they do provide insight into the potential host range of this virus.

Given the continuing circulation of the MERS-CoV virus and the associated high case fatality rate, the search for an animal infection/disease model is of utmost importance for our understanding of the pathogenesis of this virus and for the development of effective countermeasures.
